# A Comparison of the Current N2 Classification and a Modified N2 Categorization in TNM Staging of Esophageal Cancer Patients

**DOI:** 10.3389/fonc.2020.561363

**Published:** 2021-01-19

**Authors:** Kexing Xi, Hui Yu

**Affiliations:** ^1^ Department of Thoracic Surgery, Sun Yat-sen University Cancer Center, Guangzhou, China; ^2^ State Key Laboratory of Oncology in South China, Collaborative Innovation Center for Cancer Medicine, Guangzhou, China

**Keywords:** esophageal cancer, prognosis, SEER database, American Joint Committee on Cancer, N category

## Abstract

**Objective:**

To compare the effectiveness of the current N classification and a modified N2 categorization in TNM staging of esophageal cancer (EC) patients.

**Methodology:**

A total of 2753 EC patients were enrolled in the study: 2283 EC patients from the Surveillance, Epidemiology, and End Results (SEER) database and 470 separate Chinese patients were used to verify the results of the SEER database. X-tile software was employed to determine the optimal cutoff points of the number of metastatic lymph nodes (LNs) in the N2 category. Univariate and multivariate Cox regression analyses were performed to identify the survival risk factors.

**Result:**

Patients in the N2 category were divided into two groups based on the number of metastatic LNs. Patients with three and four metastatic LNs were categorized as N2a, while those with five and six metastatic LNs were categorized as N2b. The 3-year overall survival (OS) rate in the SEER database was 71.5%, 42.3%, 23.6%, 17.2%, and 10.7% for patients with N0, N1, N2a, N2b, and N3, respectively (*P*<0.001). Furthermore, a separate Chinese cohort was enrolled to validate the revised N2 category. Additionally, the 3-year OS rate was 71.5%, 42.3%, 23.6%, 17.2%, and 10.7% for patients with N0, N1, N2a, N2b, and N3, respectively (*P*<0.001).

**Conclusion:**

The current N2 category should be further divided into two groups (N2a and N2b) to provide more accurate prognosis information that could further help in developing personalized therapeutic strategies.

## Introduction

Esophageal cancer (EC) is a common cause of cancer-related death across the world (1, 2). The prognosis of EC patients is still poor; it has a reported 5-year overall survival of less than 30% (3). Lymph node (LN) status is an important and powerful prognostic factor for EC patients (4). Accurate lymph node staging is therefore important for evaluating the prognosis of EC patients as well as developing effective treatment strategies (5, 6).

According to the American Joint Committee on Cancer (AJCC) and the International Union Against Cancer (UICC) classification system, N is categorized into N0 (0), N1 (1~2), N2 (3~6), and N3 (≥7). This is based on the number of positive LNs (7). The system is invaluable in providing useful prognosis information for EC patients in clinical practice. The system generally defines patients with 3~6 positive LNs in the same N category (N2), however, thus not factoring in the effects of varying quantities of metastatic LNs within the category.

Previous studies report that increased numbers of metastatic LNs are related to poor survival in EC patients (8–10). Despite these reports, the N2 category of the current classification system is not fully annotated, thereby making it ineffective for more accurate evaluation of the prognosis of EC patients.

Herein, EC patients, clinicopathological data were sourced from the Surveillance, Epidemiology, and End Results (SEER) database and used for accurate LN staging. A modified N2 category was also proposed. The results were externally verified using a separate Chinese cohort.

## Materials and Methods

### Patient Selection

**Figure 1:**
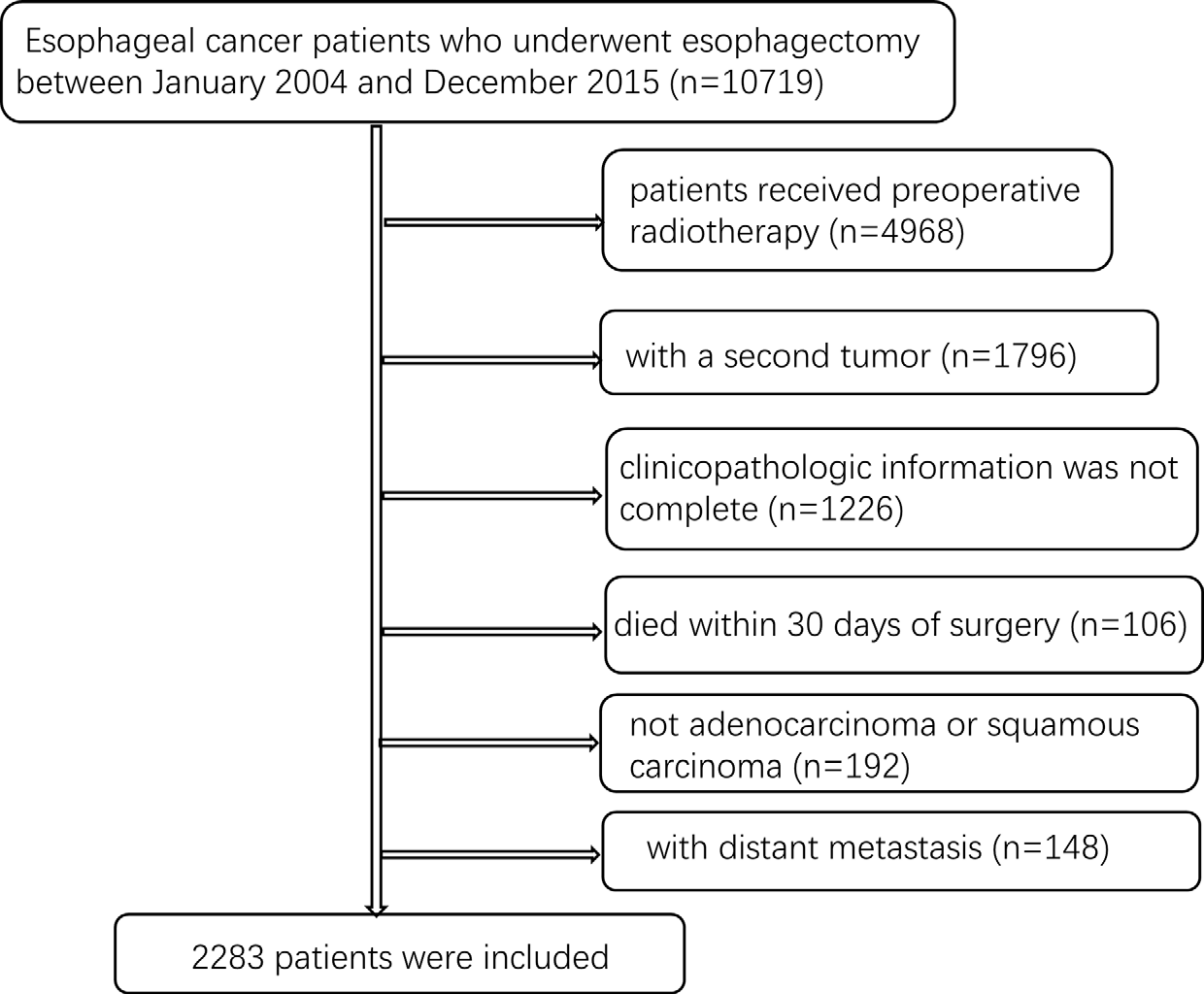
Selection flow for eligible patients in the SEER database.

In the first stage of patient selection, EC patients who underwent esophagectomy between January 2004 and December 2015 based on the SEER database search were included in the study. However, patients who received preoperative radiotherapy and those with a second tumor were excluded from the study. Those whose clinicopathologic information was not complete as well as those whose histology was not adenocarcinoma or squamous carcinoma were also excluded. This was also the case for patients who died within 30 days after surgery, were less than 18 years old, and those with distant metastasis. Finally, 2283 patients in SEER database were included for further analysis (**Figure 1**).

In the second stage of patient selection, EC patients who underwent radical resection at Sun Yat-sen University Cancer Center in Guangzhou (Guangdong, China) between January 2005 and December 2010 were included in the study. The exclusion criteria used were like the ones used in the first stage of patient selection with only a few additions. At this stage, patients with fewer than 15 LNs were examined; those without positive LNs as well as those who had received preoperative chemotherapy were excluded. Finally, 470 patients were included for further analysis. The pathologic diagnosis for all patients was based on the eighth edition of the AJCC tumor-node-metastasis (TNM) classification. Follow-up of patients were done up to July 1, 2015. This study was approved by the Ethics Committee of Sun Yat-sen University Cancer Center. All patients gave a written informed consent prior to the study.

### Statistical Analysis

The X-tile software (Version 3.6.1, Copyright Yale University 2003) was employed to determine the optimal cutoff points of the number of metastatic LNs in the N2 category using the minimum *p* value. In the same line, the Kaplan–Meier method and log-rank tests were used in the survival analysis. Additionally, univariate and multivariate Cox regression analyses were performed to identify the survival risk factors. Statistical analyses were carried out using SPSS v25.0 (IBM, Armonk, NY, USA). *P* values less than 0.05 indicated that there were significant differences between groups.

## Results

A total of 2753 EC patients were enrolled in the study: 2283 patients from the SEER database and 470 patients from a Chinese single-institution database. The distribution of clinicopathologic characteristics of all patients is summarized in **Table 1**.

**Table 1:** Distribution of clinicopathologic characteristics of all patients in both cohorts.

Variable	The SEER database n (%)	The Chinese database n (%)
Gender		
Male	1904 (83.4)	393 (83.6)
Female	379 (16.6)	77 (16.4)
Age (years)		
≤65	1288 (56.4)	384 (81.7)
>65	995 (43.6)	86 (18.3)
Race		
White	2039 (89.3)	
Black	123 (5.4)	
Others	121 (5.3)	
Marital status		
Single	349 (15.3)	
Married	1456 (63.8)	
Widowed/divorced	372 (16.3)	
Others	106 (4.6)	
Tumor size (cm)		
≤3	1078 (47.2)	84 (17.9)
>3	851 (37.3)	386 (82.1)
Unknown	354 (15.5)	
Tumor site		
Upper	58 (2.5)	34 (7.2)
Middle	271 (11.9)	297 (63.2)
Lower	1730 (75.8)	139 (29.6)
Overlapping	65 (2.8)	
Unknown	159 (7.0)	
Surgical approach		
Through left chest		270 (57.4)
Through right chest		200 (42.6)
Anastomosis		
Hand-sewn		73 (15.5)
Stapled		397 (84.5)
Differentiation		
Well	247 (10.8)	76 (16.2)
Moderate	989 (43.3)	236 (50.2)
Poor	867 (38.0)	158 (33.6)
Unknown	180 (7.9)	
Histology		
Adenocarcinoma	1814 (79.5)	6 (1.3)
Squamous cell carcinoma	469 (20.5)	464 (98.7)
pT status		
T1	1069 (46.8)	19 (4.0)
T2	335 (14.7)	65 (13.8)
T3	800 (35.0)	350 (74.5)
T4	79 (3.5)	36 (7.7)
pN status		
N0	1474 (64.6)	0 (0)
N1	447 (19.6)	246 (52.3)
N2	237 (10.4)	173 (36.8)
N3	125 (5.5)	51 (10.9)
Number of examined LNs		
<15	1277 (55.9)	0 (0)
≥15	1006 (44.1)	470 (100)
Adjuvant radiation		
Yes	439 (19.2)	13 (2.8)
No	1844 (80.8)	457 (97.2)
Chemotherapy		
Yes	727 (31.8)	140 (29.8)
No	1556 (68.2)	330 (70.2)

LNs, lymph nodes.

**Figure 2:**
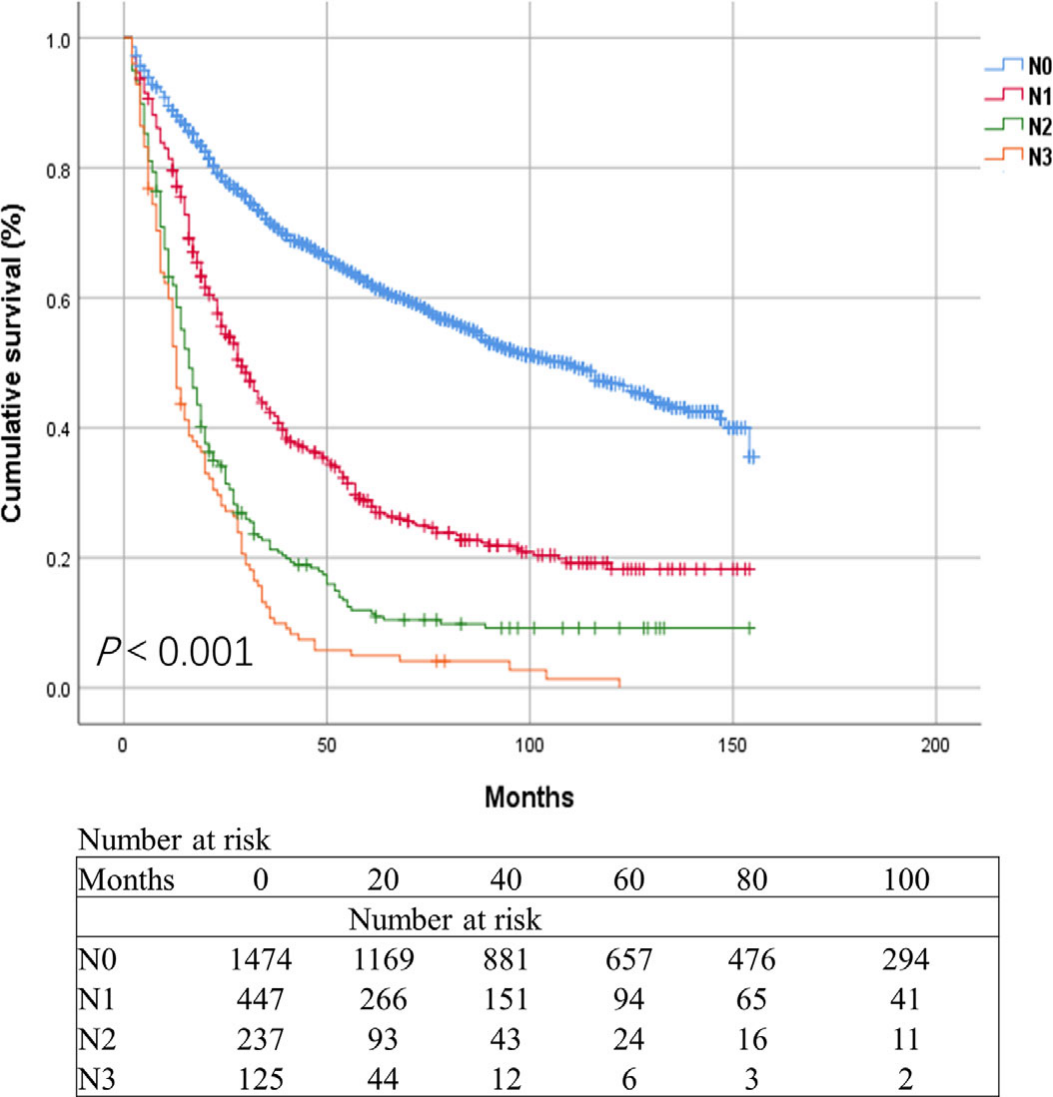
Kaplan-Meier survival curves for esophageal cancer patients in the SEER database based on the current N categories.

There were significant differences in the survival rates between the different current N groups of the data from 2283 patients obtained from the SEER database. The overall survival (OS) rate after 3 years was 71.5% for N0 patients compared with 42.3%, 21.3%, and 10.7% for those with N1, N2, and N3, respectively *(P*<0.001, **Figure 2**). In the univariate and multivariate survival, analyses revealed that the current N category was a risk factor for survival (*P*<0.001, **Table 2**).

**Table 2:** Cox univariate and multivariate analyses of prognostic factors for 2283 esophageal cancer patients in the SEER database.

Variable	Univariate analysis		Multivariate analysis	
	HR (95% CI)	P	HR (95% CI)	P
Gender		0.522		
Male	Reference			
Female	1.049 (0.906~1.215)			
Age (years)		<0.001		<0.001
≤65	Reference		Reference	
>65	1.385 (1.241~1.545)		1.428 (1.274~1.600)	
Race		<0.001		0.003
White	Reference		Reference	
Black	1.572 (1.261~1.960)	<0.001	1.129 (0.891~1.431)	0.316
Others	0.866 (0.667~1.125)	0.281	0.665 (0.507~0.872)	0.003
Marital status		0.008		
Single	Reference			
Married	0.952 (0.813~1.116)	0.544		
Widowed/divorced	1.220 (1.008~1.476)	0.041		
Others	0.907 (0.669~1.230)	0.531		
Tumor size (cm)		<0.001		
≤3	Reference			
>3	1.998 (1.776~2.247)	<0.001		
Unknown	0.852 (0.714~1.017)	0.076		
Tumor site		0.008		0.048
Upper	Reference		Reference	
Middle	1.108 (0.778~1.580)	0.569	0.992 (0.693~1.419)	0.964
Lower	0.828 (0.596~1.151)	0.261	0.774 (0.551~1.089)	0.141
Overlapping	0.933 (0.595~1.463)	0.763	0.720 (0.456~1.138)	0.160
Unknown	0.845 (0.575~1.244)	0.394	0.913 (0.616~1.351)	0.648
Differentiation		<0.001		<0.001
Well	Reference		Reference	
Moderate	1.832 (1.464~2.293)	<0.001	1.348 (1.074~1.693)	0.010
Poor	2.631 (2.105~3.289)	<0.001	1.666 (1.323~2.098)	<0.001
Unknown	1.019 (0.744~1.396)	0.905	1.207 (0.878~1.658)	0.246
Histology		<0.001		0.001
Squamous cell carcinoma	Reference		Reference	
Adenocarcinoma	0.601 (0.531~0.682)		0.765 (0.654~0.896)	
pT status		<0.001		<0.001
T1	Reference		Reference	
T2	2.023 (1.704~2.404)	<0.001	1.734 (1.445~2.081)	<0.001
T3	4.141 (3.640~4.710)	<0.001	3.140 (2.680~3.678)	<0.001
T4	3.677 (2.820~4.793)	<0.001	2.627 (1.972~3.499)	<0.001
pN status		<0.001		<0.001
N0	Reference		Reference	
N1	2.364 (2.066~2.706)	<0.001	1.941 (1.672~2.253)	<0.001
N2	4.091 (3.489~4.797)	<0.001	3.067 (2.560~3.675)	<0.001
N3	5.585 (4.580~6.810)	<0.001	4.118 (3.285~5.162)	<0.001
Number of examined LNs		0.003		<0.001
<15	Reference		Reference	
≥15	0.846 (0.757~0.945)		0.702 (0.627~0.787)	
Adjuvant radiation		<0.001		
Yes	Reference			
No	0.603 (0.531~0.684)			
Chemotherapy		<0.001		<0.001
Yes	Reference		Reference	
No	0.660 (0.590~0.739)		1.578 (1.378~1.805)	

LNs, lymph nodes; HR, Hazard ratio; CI, confidence interval.

**Figure 3:**
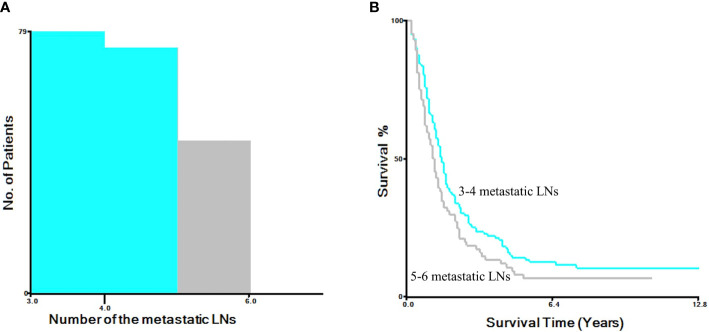
X-tile software analysis to determine the optimal cutoff points for N2 group based on the number of metastatic LNs. **(A)** The histogram of the number of the metastatic LNs. **(B)** Kaplan-Meier analysis for survival.

**Figure 4:**
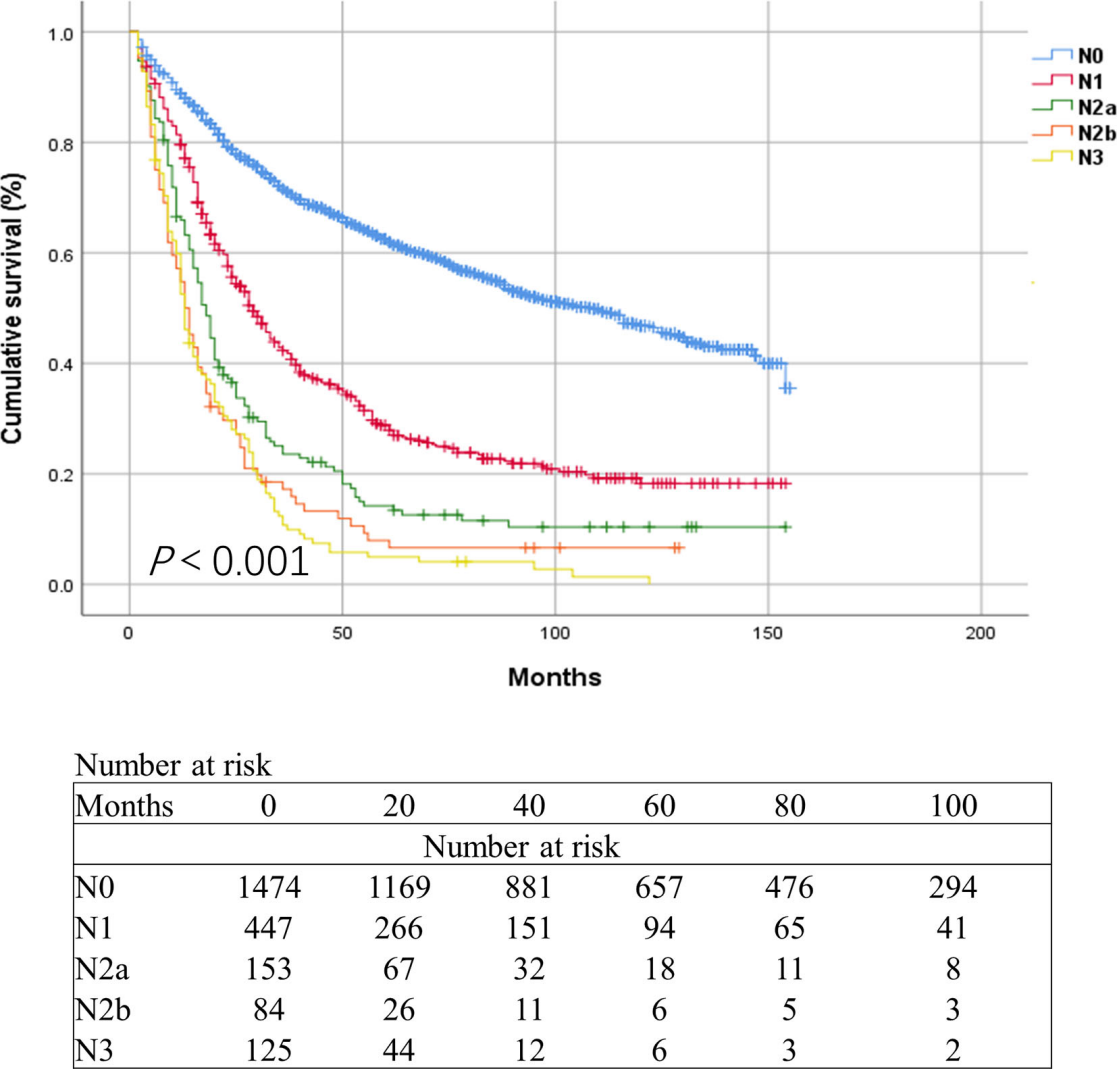
Kaplan-Meier survival curves for esophageal cancer patients of in the SEER database based on the revised N categories.

Results of the X-tile software divided patients in the current N2 category into two groups based on the number of metastatic LNs. Patients with three and four metastatic LNs were categorized as N2a, while those with five and six metastatic LNs were categorized as N2b (**Figure 3**). We firstly estimated the revised N2 category in the SEER database using Kaplan-Meier method. The 3-year OS rate was 71.5%, 42.3%, 23.6%, 17.2%, and 10.7% for patients with N0, N1, N2a, N2b, and N3, respectively (*P*<0.001, **Figure 4**). Cox univariate and multivariate regression analyses were performed to evaluate the revised N2 category. Univariate analysis results revealed that age, race, marital status, tumor size, tumor site, differentiation, histology, pT status, pN status, number of examined LNs, adjuvant radiation, and chemotherapy were significantly associated with OS. In the same line, multivariate analysis results revealed that age, race, tumor site, differentiation, histology, pT status, pN status, number of examined LNs, and chemotherapy were significantly associated with OS [all *P* values were less than 0.001 except for tumor site (*P*=0.049) and histology (*P*=0.001)] (**Table 3**).

**Table 3:** Cox univariate and multivariate analyses of prognostic factors for 2283 esophageal cancer patients based on the revised N categories in the SEER database.

Variable	Univariate analysis		Multivariate analysis	
	HR (95% CI)	P	HR (95% CI)	P
Gender		0.522		
Male	Reference			
Female	1.049 (0.906~1.215)			
Age (years)		<0.001		<0.001
≤65	Reference		Reference	
>65	1.385 (1.241~1.545)		1.427 (1.273~1.599)	
Race		<0.001		0.003
White	Reference		Reference	
Black	1.572 (1.261~1.960)	<0.001	1.132 (0.893~1.435)	0.307
Others	0.866 (0.667~1.125)	0.281	0.670 (0.511~0.880)	0.004
Marital status		0.008		
Single	Reference			
Married	0.952 (0.813~1.116)	0.544		
Widowed/divorced	1.220 (1.008~1.476)	0.041		
Others	0.907 (0.669~1.230)	0.531		
Tumor size (cm)		<0.001		
≤3	Reference			
>3	1.998 (1.776~2.247)	<0.001		
Unknown	0.852 (0.714~1.017)	0.076		
Tumor site		0.008		0.049
Upper	Reference		Reference	
Middle	1.108 (0.778~1.580)	0.569	0.989 (0.691~1.416)	0.953
Lower	0.828 (0.596~1.151)	0.261	0.773 (0.550~1.086)	0.138
Overlapping	0.933 (0.595~1.463)	0.763	0.720 (0.456~1.138)	0.159
Unknown	0.845 (0.575~1.244)	0.394	0.909 (0.614~1.346)	0.634
Differentiation		<0.001		<0.001
Well	Reference		Reference	
Moderate	1.832 (1.464~2.293)	<0.001	1.351 (1.076~1.696)	0.010
Poor	2.631 (2.105~3.289)	<0.001	1.670 (1.326~2.103)	<0.001
Unknown	1.019 (0.744~1.396)	0.905	1.210 (0.881~1.662)	0.240
Histology		<0.001		0.001
Squamous cell carcinoma	Reference		Reference	
Adenocarcinoma	0.601 (0.531~0.682)		0.766 (0.654~0.897)	
pT status		<0.001		<0.001
T1	Reference		Reference	
T2	2.023 (1.704~2.404)	<0.001	1.739 (1.448~2.087)	<0.001
T3	4.141 (3.640~4.710)	<0.001	3.132 (2.673~3.670)	<0.001
T4	3.677 (2.820~4.793)	<0.001	2.623 (1.969~3.495)	<0.001
pN status		<0.001		<0.001
N0	Reference		Reference	
N1	2.365 (2.067~2.707)	<0.001	1.942 (1.673~2.254)	<0.001
N2a	3.713 (3.070~4.492)	<0.001	2.948 (2.394~3.631)	<0.001
N2b	4.949 (3.898~6.284)	<0.001	3.299 (2.555~4.260)	<0.001
N3	5.590 (4.584~6.817)	<0.001	4.123 (3.289~5.169)	<0.001
Number of examined LNs		0.003		<0.001
<15	Reference		Reference	
≥15	0.846 (0.757~0.945)		0.702 (0.626~0.787)	
Adjuvant radiation		<0.001		
Yes	Reference			
No	0.603 (0.531~0.684)			
Chemotherapy		<0.001		<0.001
Yes	Reference		Reference	
No	0.660 (0.590~0.739)		1.577 (1.378~1.805)	

LNs, lymph nodes; HR, Hazard ratio; CI, confidence interval.

**Figure 5:**
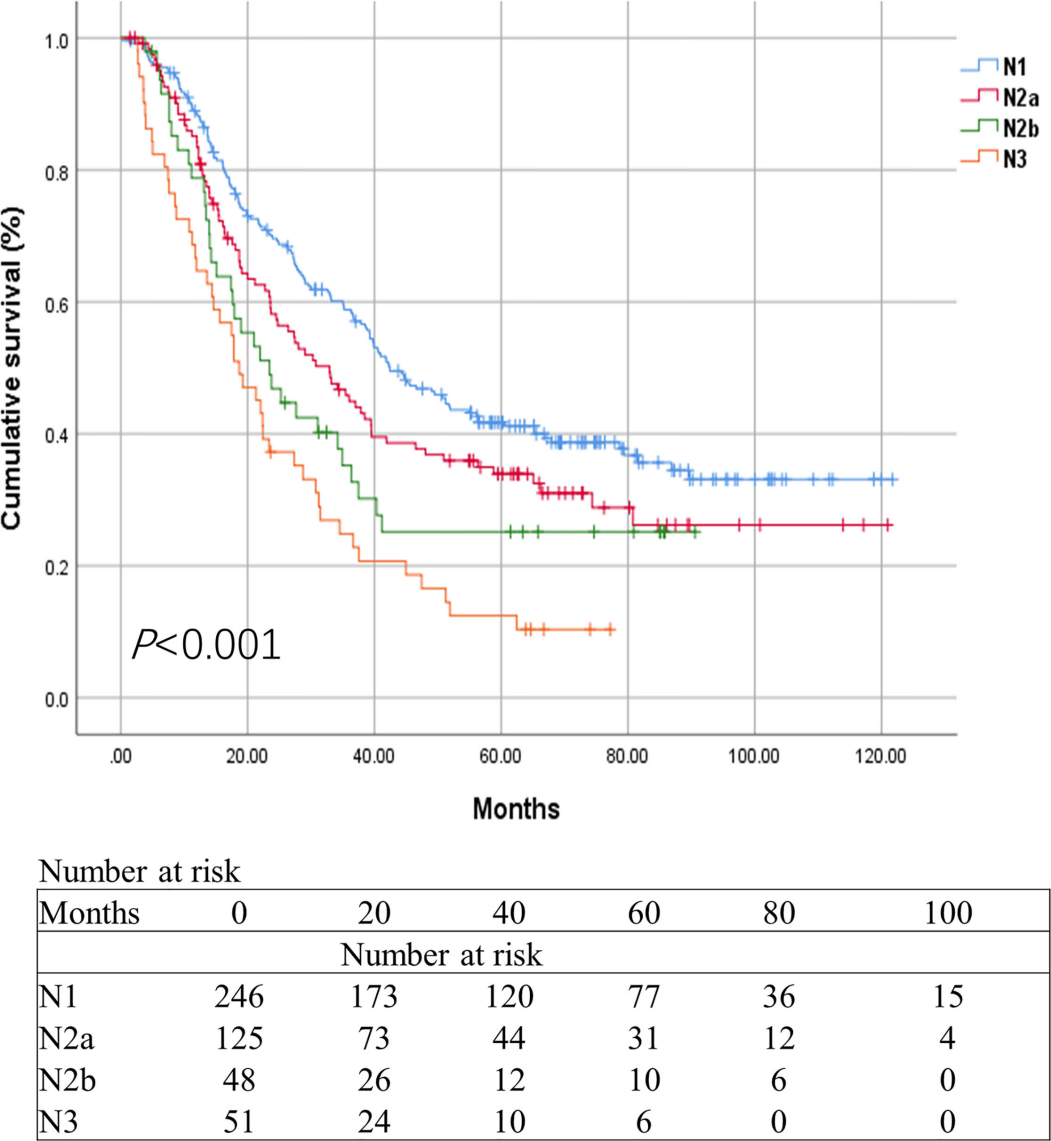
Kaplan-Meier survival curves for esophageal cancer patients in the Chinese cohort based on the revised N categories.

These results were validated using a separate Chinese cohort. In the group, patients in the N1 category had significantly better survival than those in the N2a, N2b, and N3 categories. The 3-year OS rate was 58.8%, 45.8%, 35.2%, and 24.8%, respectively. The 5-year OS rate was 41.7%, 33.9%, 25.1%, and 12.4%, respectively (*P*<0.001) (**Figure 5**). Multivariate analysis of the OS demonstrated that the pT status, pN status, and age were independent prognostic factors (*P*<0.001, <0.001, and 0.040, respectively). Tumor size was not associated with the OS (*P*=0.570). Both univariate and multivariate analyses revealed that there were significant differences in survival rate between N2a group and N2b group (**Table 4**). The revised N2 category was significantly associated with the survival of patients in the SEER database and the Chinese cohort.

**Table 4:** Cox univariate and multivariate analyses of prognostic factors for esophageal cancer patients based on the revised N categories in the Chinese cohort.

Variable	Univariate analysis		Multivariate analysis	
	HR (95% CI)	P	HR (95% CI)	P
Gender		0.606		
Male	Reference			
Female	0.925 (0.686~1.246)			
Age (years)		0.032		0.040
≤65	Reference		Reference	
>65	1.352 (1.026~1.782)		1.340 (1.013~1.774)	
Tumor size (cm)		0.028		
≤3	Reference			
>3	1.399 (1.037~1.886)			
Tumor site		0.471		
Upper	Reference			
Middle	0.825 (0.552~1.234)	0.350		
Lower	0.763 (0.494~1.177)	0.221		
Surgical approach		0.288		
Through left chest	Reference			
Through right chest	0.884 (0.704~1.110)			
Anastomosis		0.300		
Hand-sewn	Reference			
Stapled	0.855 (0.636~1.150)			
Differentiation		0.408		
Well	Reference			
Moderate	1.252 (0.896~1.749)	0.188		
Poor	1.227 (0.862~1.746)	0.257		
Histology		0.616		
Squamous cell carcinoma	Reference			
Adenocarcinoma	1.288 (0.480~3.456)			
pT status		<0.001		<0.001
T1	Reference		Reference	
T2	1.871 (0.871~4.019)	0.108	1.844 (0.855~3.981)	0.119
T3	2.489 (1.229~5.040)	0.011	2.241 (1.102~4.556)	0.026
T4	5.260 (2.410~11.484)	<0.001	5.011 (2.282~11.002)	<0.001
pN status		<0.001		<0.001
N1	Reference		Reference	
N2a	1.306 (0.994~1.715)	0.055	1.274 (0.968~1.676)	0.084
N2b	1.651 (1.136~2.401)	0.009	1.514 (1.038~2.209)	0.031
N3	2.493 (1.780~3.492)	<0.001	2.630 (1.869~3.703)	<0.001
Adjuvant radiation		0.467		
Yes	Reference			
No	1.321 (0.624~2.795)			
Adjuvant chemotherapy		0.054		
Yes	Reference			
No	1.283 (0.995~1.655)			

LNs, lymph nodes; HR, Hazard ratio; CI, confidence interval.

## Discussion

The LNs status is an important prognostic factor of survival for EC patients (5, 11, 12). Current N categories are based mainly on the number of positive LNs. However, the current staging system categorizes patients with between three and six metastatic LNs as N2. As such, it does not factor in the effects of different number of metastatic LNs within the patients categorized in this subgroup.

Herein, patients in the current N2 category were divided into two groups (N2a and N2b) based on the results of X-tile analysis. The software employs a cut-point selection to divide a single cohort into a training and validation subset for *P* value evaluation, and further determines the optimal cut-off points using the minimum *P* value (13). The revised N category was found to be significantly associated with the OS of the patients. These results were further validated using a separate Chinese cohort under tougher criterions to improve the effectiveness of the validation.

The current TNM staging system for EC stratifies patients into different N groups, i.e., N0 to N3. Herein, there were significant differences in survival rates between patients (SEER database) in different N groups. Higher N categories had poorer prognoses compared to lower N categories. These results were consistent with previous studies (4, 14). Similar results were obtained in the revised N category, i.e., N2a patients had a better OS rate compared with N2b patients (3-year survival rate: 23.6% vs 17.2%). Similarly, there were significant differences in the survival rate of the Chinese cohort categorized in N1, N2a, N2b, and N3 groups. Cognizant to this, the revised N2 category was more effective, providing more prognosis information than the current N2 category. The results further revealed that N2b patients might require more subsequent intense treatment such as the increased chemotherapy doses than N2a patients. Though some new prognostic parameters, such as the LN ratio, have been reported, the optimal thresholds of LN ratio vary in different studies, thereby limiting its clinical application (15, 16). As such, the N category (pN) based on the number of metastatic LNs was more simple and easier to promote in hospitals of various levels.

pT status, pN status, and age were found to be independent EC prognostic factors in both the SEER and Chinese single-center database. These results were consistent with previous literature (17, 18). In the SEER database, differentiation and histology were significantly associated with OS of patients. However, these variables were not independent risk factors of survival in the Chinese database. This might be attributed to the small size (only six adenocarcinoma patients) of the Chinese database. The differences in the two populations also brought some bias. In addition, patients in the SEER database with 15 of more LNs examined had a better survival compared with those with less than 15 LNs examined. These results were consistent with previous studies. Such as Ajani et al. had recommended that at least 15 LNs should be examined for EC patients who underwent esophagectomy (4, 19). The SEER database collected data from different institutions and there is no consensus on the optimal number of examined LNs for EC patients in previous years. Different institutions or surgeons had different opinions and habits, which might result in a variety in the number of LN examined. Herein, we collected both patients with ≥15 and <15 LNs examined in the first stage. Additionally, more cases were enrolled for analysis, and the survival difference between patients with ≥15 and <15 LNs examined could be evaluated.

Herein, patients were enrolled from two databases, thereby making the revised N2 category more representative and reliable. Approximately 80% of the patients in the SEER database had been diagnosed with adenocarcinoma while 99% of patients in the Chinese cohort had been diagnosed with squamous cell carcinoma. The revised N2 category proved to be effective in both cohorts, strongly suggesting that it was appropriate for different EC histological types.

Nevertheless, this study was limited by several factors. It was a retrospective study, and the bias was inevitable. There was only a small number of esophageal adenocarcinoma patients in the Chinese cohort. Moreover, some vital information, such as the detail of chemotherapy and data of tumor recurrence, was not available in the SEER database. As such, a large, multi-institutional study should be conducted in the future to further address these limitations.

Evidently, the eighth edition AJCC N classification provides postoperative survival information for esophageal cancer patients. However, the provided information is insufficient for a more accurate prognosis. As such, the current N2 category should be further divided into two groups (N2a and N2b) to provide more accurate prognosis information that could further help in developing personalized therapeutic strategies.

## Data Availability Statement

The datasets presented in this study can be found in the SEER database (2004–2015). Other datasets generated for this study are available on request to the corresponding author.

## Ethics Statement

The studies involving human participants were reviewed and approved by the Institute Research Medical Ethics Committee of Sun Yat-sen University Cancer Center. The patients/participants provided their written informed consent to participate in this study.

## Author Contributions

(I) Conception and design: KX. (II) Administrative support: HY. (III) Provision of study materials or patients: KX, HY. (IV) Collection and assembly of data: KX, HY. (V) Data analysis and interpretation: KX. (VI) Manuscript writing: All authors. All authors contributed to the article and approved the submitted version.

## Conflict of Interest

The authors declare that the research was conducted in the absence of any commercial or financial relationships that could be construed as a potential conflict of interest.
